# RetroRanker: leveraging reaction changes to improve retrosynthesis prediction through re-ranking

**DOI:** 10.1186/s13321-023-00727-7

**Published:** 2023-06-08

**Authors:** Junren Li, Lei Fang, Jian-Guang Lou

**Affiliations:** 1 College of Chemistry and Molecular Engineering, Peking University, No. 5 Yiheyuan Road, Beijing, 100871 China; 2 Microsoft Research Asia, Building 2, No. 5 Dan Ling Street, Beijing, 100080 China

**Keywords:** Retrosynthesis, Re-ranking, Graph neural networks

## Abstract

Retrosynthesis is an important task in organic chemistry. Recently, numerous data-driven approaches have achieved promising results in this task. However, in practice, these data-driven methods might lead to sub-optimal outcomes by making predictions based on the training data distribution, a phenomenon we refer as frequency bias. For example, in template-based approaches, low-ranked predictions are typically generated by less common templates with low confidence scores which might be too low to be comparable, and it is observed that recorded reactants can be among these low-ranked predictions. In this work, we introduce RetroRanker, a ranking model built upon graph neural networks, designed to mitigate the frequency bias in predictions of existing retrosynthesis models through re-ranking. RetroRanker incorporates potential reaction changes of each set of predicted reactants in obtaining the given product to lower the rank of chemically unreasonable predictions. The predicted re-ranked results on publicly available retrosynthesis benchmarks demonstrate that we can achieve improvement on most state-of-the-art models with RetroRanker. Our preliminary studies also indicate that RetroRanker can enhance the performance of multi-step retrosynthesis.

**Supplementary Information:**

The online version contains supplementary material available at 10.1186/s13321-023-00727-7.

## Introduction

Organic chemistry is a discipline primarily focused on studying and creating organic compounds. Retrosynthesis, which aims to propose a list of candidate reactants that likely lead to a given product, is a critical task in organic chemistry. Early approaches in retrosynthesis planning typically involve manually analyzing the target molecule and subsequently dividing it into synthesizable precursors step by step, which requires extensive chemistry knowledge [1]. The first computer program designed to assist in proposing the retrosynthesis plan was developed in the 1960s [2, 3], primarily addressing the problem of recommending and building synthetic templates. As the number of chemical reaction rules increases, it becomes costly to construct a reasonably effective expert system with comprehensive organic chemistry knowledge [4–6].

With recent rapid advancements in artificial intelligence, numerous fully data-driven approaches have demonstrated promising results in single-step retrosynthesis prediction, which can be broadly classified into two categories: template-based and template-free. For template-based approaches, they first extract reaction templates from a reaction database and then employ a ranking or classification model to select potentially correct templates based on molecular similarity for a given product [7–9]. For template-free approaches, chemical reactions are typically represented as SMILES (Simplified Molecular-Input Line-Entry System) strings [10, 11], and the retrosynthesis task is formulated as a text generation problem using encoder-decoder architectures [12–15]. In this context, the encoder encodes the molecular sequence [6, 13, 16] or graph [17, 18] as high dimensional vectors, and the decoder predicts the output sequence based on the contextual representation from the encoder [5].

**Fig. 1:**
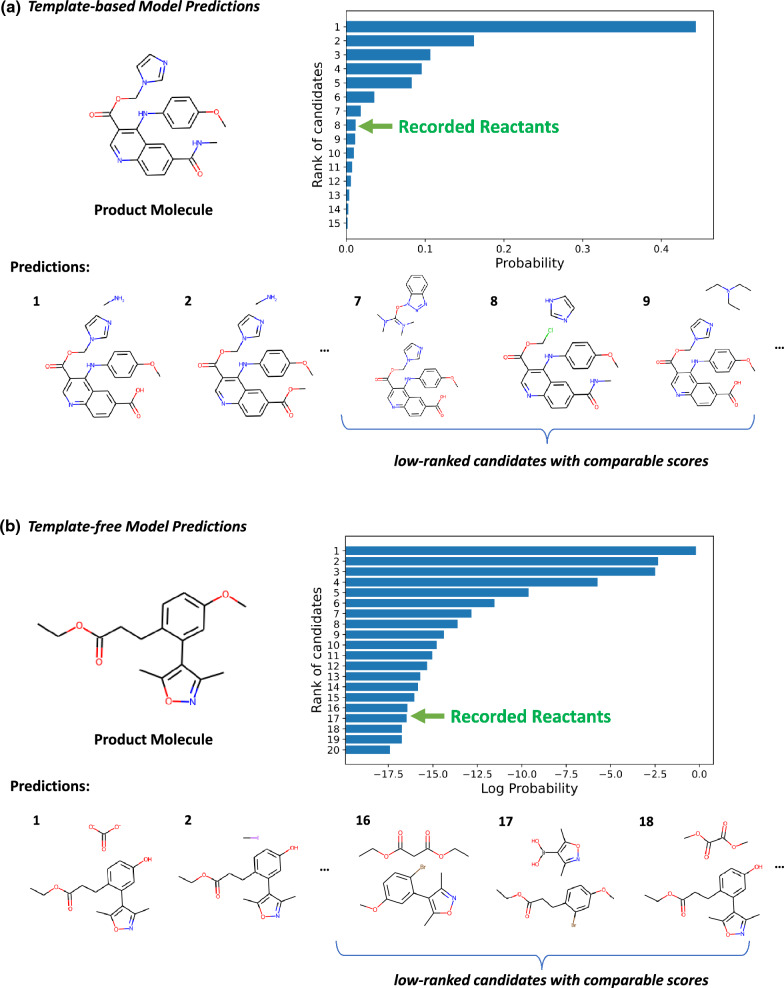
Predictions of template-based (**a**) and template-free (**b**) approaches. The bar charts show the probabilities and logarithmic probabilities of each prediction. We show top-ranked predictions and predictions around the recorded reactants. The ranking scores of low-ranked results are comparable, which are all significantly lower than that of top-ranked entries

In both template-based and template-free approaches, the output are a list of ranked reactants. The ranking in template-based approaches primarily relies on the template score and reactant score [8], whereas in template-free approaches, it depends on the probability of generating the sequence of reactants’ SMILES during beam search [6, 13, 16]. Data-driven approaches may produce sub-optimal results by making predictions based on the training data distribution, which we interpret as frequency bias. In template-based approaches, top-ranked predictions are typically generated by common templates with relatively high scores, while low-ranked ones are produced by less common templates with low confidence scores, which might be too low to be comparable. Similarly, template-free methods tend to decode output SMILES with common “decoding patterns” [19], i.e., the model attempts to apply frequent synthesis patterns (on SMILES) learned from the training data to a given product. It has been observed that the recorded reactants in patent pathways can be among those low-ranked predictions. We present two examples in Fig. 1.

In Fig. 1(a), the product molecule has multiple functional groups that can be synthesized during reactions. It can be obtained by cleaving the ester group, the amide group, or the secondary amine of the given product, with each of these sites corresponding to multiple possible precursors. The top-1 result^1^ of the standard template-based approach [20] is a common amidation reaction that combines the carboxylic acid group with a methylamine. The top-ranked predictions may have selectivity issues (a new amide could be formed at the other carboxyl group in the top-1 prediction), and the reactants might also be challenging to synthesize (1 and 2). The recorded set of reactants is ranked at 8, which is more feasible when compared to other predictions. However, its ranking score is comparable to its neighbors (7 and 9) and is significantly lower than the top-ranked ones. For template-free models, we display the predictions and the logarithmic probabilities of generating the SMILES strings by Augmented Transformer [14] in Fig. 1(b). The top-1 prediction will not react, and the reactants in the second prediction may be difficult to obtain. It appears that the model is attempting to apply frequent synthesis patterns at SMILES level to the given product. The recorded set of reactants is at a low rank, and its ranking score is comparable to its neighbors, which is also significantly lower than top-ranked ones. The two examples demonstrate that ranking predictions by those relatively low confidence scores can be unreliable and may pose problems from a chemical perspective.

In order to filter out chemically unreasonable predictions, Segler et al. [21] trained a classifier to evaluate the feasibility of each prediction reacting to the given product based on molecular fingerprints. Schwaller et al. [13] built a round-trip prediction using the forward synthesis model, which might also have the frequency bias as previously mentioned. Sun et al. [22] tackled the retrosynthesis problem from an energy-based perspective and trained a dual model to combine forward (reaction prediction) and backward (retrosynthesis) directions to rank the predicted reactants. Lin et al. [23] proposed to re-rank the predictions using energy-based models to improve the performance of several single-step models. Their ranking models are mainly based on molecule graphs [23] or fingerprints [21], which do not incorporate potential reaction changes.

In this paper, we propose RetroRanker, a method designed to mitigate the frequency bias of existing data-driven approaches by re-ranking predictions with low confidence scores. RetroRanker is built upon graph neural networks (GNN) leveraging chemical features from both molecular graphs and potential reactions. These chemical features are independent of the aforementioned text generation or extracted templates, offering complementary information beyond the existing frequency bias. We demonstrate improvements over existing state-of-the-art models on both USPTO-50K [24] and USPTO-full [25, 26] datasets. Our preliminary studies also indicate that improved performance can be achieved in multi-step retrosynthesis using RetroRanker.

## Methods

**Fig. 2:**
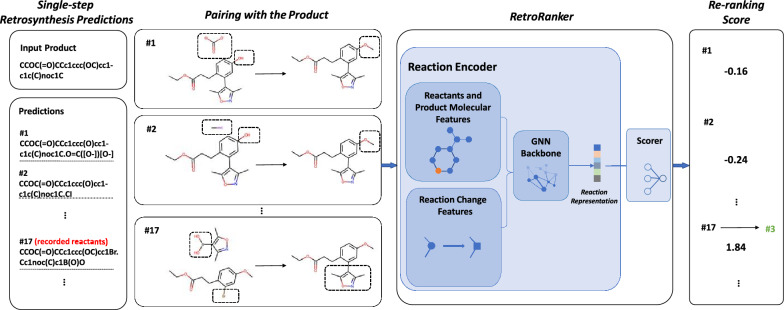
Overview of re-ranking with RetroRanker. Each prediction from the single-step retrosynthesis model is paired with the product as input to RetroRanker (the potential reaction changes are highlighted in dashed boxes). RetroRanker consists of a reaction encoder and a scorer module. The reaction encoder takes the molecular features and the potential reaction changes as input, which are further updated with GNN backbones. The re-ranking score is calculated based on the high-dimensional representation from GNN

Given a product molecule and a list of corresponding predictions, where each entry is a set of reactants, RetroRanker aims to re-rank the low-ranked entries, which usually have low confidence scores. Following learning-to-rank techniques [27] in information retrieval and machine learning, we designed RetroRanker as a pairwise ranking model, with the training objective that recorded reactants have a higher score than non-recorded predictions (Fig. 2).

Each prediction from the single-step retrosynthesis model is paired with the product as input to RetroRanker, which consists of a reaction encoder and a scorer module. The reaction encoder takes the reactants and product’s molecular features and their potential reaction changes as input, which are further updated with GNN backbones. The re-ranking score is calculated based on the high-dimensional representation from GNN. The final ranking is based on the original rank and the score of RetroRanker, which will be introduced later.

### Reaction encoder

In the context of this paper, the term “reaction” primarily refers to the potential reaction occurring between the predicted reactants and the given product. It is important to note that some reactants may not undergo a reaction in the real world. RetroRanker can be viewed as a tool for ranking the feasibility of each set of predicted reactants in achieving the given product during potential reactions. Recently, various methods have been developed to learn representations of chemical reactions. DRFP [28] takes SMILES strings of reactants and product as input, and calculates reaction fingerprints based on the differences between circular substructures of reactants and products. Tavakoli et al. [29] proposed rxn-hypergraph, which utilizes hypernodes over molecular graphs to learn representations at reaction level.

**Fig. 3:**
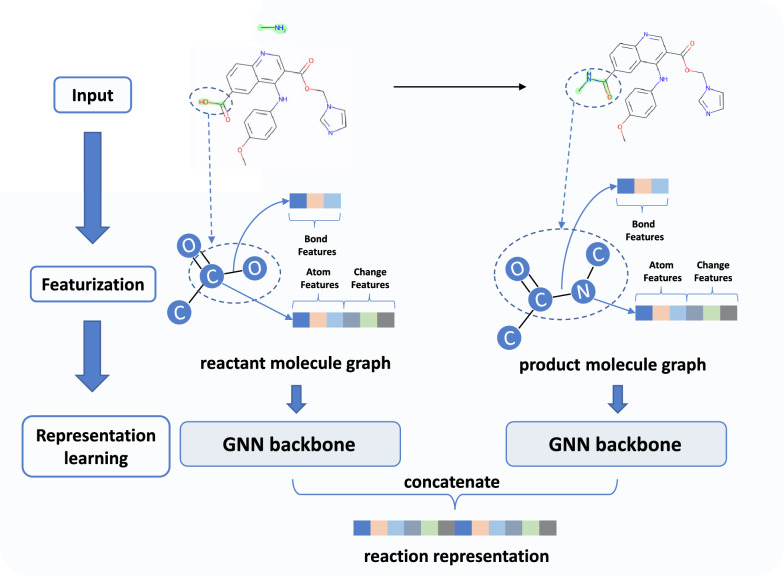
Overview of the reaction encoder. The encoder takes the predicted reactants and the given product as input. The features of each atom are composed of its molecule-level features (e.g., atom degree, bond order) and reaction change features (e.g., number of reacted atoms in the neighborhood). After featurization, the reactant molecular graph and the product molecular graph are updated by GNN backbones, respectively. The reaction representation is obtained by concatenating the GNN output of the reactant and product

The reaction encoder in RetroRanker encodes both molecular features and potential reaction change features. All features are designed at atom or bond level on the molecular graphs of reactants and product, as illustrated in Fig. 3.

Following [30], the molecular features include typical atom and bond features, such as atom degree and bond order. Potential reaction changes correspond to specific areas in molecular graphs of the reactants and the product. The reaction sites vary among different reactant-product pairs. These potential reaction changes are critical because chemically unreasonable changes indicate that the reaction will not occur in the real world. Thus, we incorporate reaction changes as input features in the reaction encoder. For each reactant-product pair, we first map the atoms in reactants and product with RXNMapper [31], and locate the reaction site in the reactants and the changed area in the product based on the mapped atoms. We extract reaction change features, such as the number of reacted atoms in the neighborhood and the number of atoms in leaving groups during the potential reaction. Note that for different reactant-product pairs, the reaction change features on the product molecule are different, even though the molecular features are the same. Please refer to the Additional file 1: Section 1 for a complete list of molecular features and reaction change features used in this paper. With both molecular features and reaction change features, we believe subtle differences among different reactant-product pairs are captured by the reaction encoder.

With atom- and bond-level features, it is natural to learn representations of reactions leveraging GNN architectures, which have shown promising results in tasks where 2D molecular graphs serve as the input [32, 33]. We choose two representative GNN architectures as backbones: AttentiveFP [33] and Graphormer [34]. It is worth noting that other variants of GNNs should also work here. AttentiveFP [33] is designed to capture chemically non-local effects (e.g., conjugated effect) among nodes on 2D molecular graphs because, in vanilla message-passing neural networks (MPNN), the interactions between nodes decay rapidly as the distance increases. AttentiveFP is capable of capturing long-range interactions through the graph attention mechanism [35], and it achieves promising performance in predicting molecular properties [33, 36]. As Transformers have achieved great success in sequence-based input tasks in natural language processing, many researchers have tried to extend the architecture to handle graph data [37, 38]. Graphormer [34] introduces centrality encoding and spatial encoding to model graph structures in Transformers. The centrality encoding adds the degree of each node as input features to encode the node importance in the graph. The spatial encoding encodes the spatial relation between every two nodes based on the shortest path. Graphormer achieves state-of-the-art performance on various graph prediction tasks [34, 39]. Please refer to the original papers [33, 34] for details about AttentiveFP and Graphormer. In the reaction encoder, we use two independent GNN backbones to learn representations of reactants and product, respectively. The reaction representation is obtained by concatenating the GNN output of the reactants and the product. The scorer in RetroRanker is a neural network of two linear layers with the reaction representation as input. We construct training data based on predictions of the given single-step retrosynthesis model, with each prediction paired with the product as input to RetroRanker. During the training process, the objective is to ensure that recorded reactants have a higher score than non-recorded predictions. We use the label smoothed cross-entropy loss to train the model. During inference, we use the output scores from RetroRanker for re-ranking.

### Re-ranking strategies

Our goal is to mitigate the frequency bias in predictions of existing data-driven models, i.e., we aim to re-rank those low-ranked predictions with relatively low confidence scores. Therefore, the requirement for re-ranking is that top-ranked predictions should be given more respect. This motivates us to design the following two re-ranking strategies.

- Strategy 1 (*S*1): We lower the rankings of predictions whose RetroRanker scores are among the bottom of ratio *p*, re-ordering them based on ranking scores from RetroRanker. The rankings of top *k* predictions are preserved. For example, $p=70\%$, $k=5$ means that we lower the rankings of predictions whose RetroRanker scores are among the bottom $70\%$, i.e., these predictions are moved to the end of the ranked list following the ranking scores by RetroRanker, and the rankings of top 5 predictions remain unchanged.
- Strategy 2 (*S*2): The final ranking is based on the sum of the original ranking and the new ranking calculated by *S*1. Compared to *S*1, The original ranking weighs more in this strategy.

For both strategies *S*1 and *S*2, the original rankings are taken into account for the final ranking. Both two strategies are flexible, as we can tune the parameters *p* and *k* (denoted as *S*1(*p*, *k*) or *S*2(*p*, *k*)) to achieve improved performance under certain requirements. The parameters of *p* and *k* can be empirically set or can be tuned with grid search. Please refer to Additional file 1: Section 4 for more discussions on the two parameters, we also give several illustrative examples to illustrate the re-ranking process of the two strategies in Additional file 1: Table S2.

### Data and settings

**Table 1:** Single-step retrosynthesis models and datasets

Model	Dataset	Pairs	Model type			
			Template-based	Template-free	Graph	Sequence
RetroXpert [40]	USPTO-50k*	1.9 million		$\checkmark$	$\checkmark$	$\checkmark$
GLN [26]	USPTO-50k*	1.8 million	$\checkmark$		$\checkmark$	
R-SMILES [15]	USPTO-50k	1.2 million		$\checkmark$		$\checkmark$
AT [14]$^\dag$	USPTO-full	12.3 million		$\checkmark$		$\checkmark$
R-SMILES [15]	USPTO-full	16.3 million		$\checkmark$		$\checkmark$

$^{*}$ Duplicated reactions are further removed, the sizes of train/valid/test are slightly changed to 39713/4989/5005 [23]

$^{\dag }$ AT is for Augmented Transformer

The USPTO dataset is a well-adopted reaction dataset for single-step retrosynthesis prediction, which contains organic reactions extracted from US Patent and Trademark Office (USPTO)-granted patents [5, 25, 41]. For reactions extracted from patents [25] with multiple products, Dai et al. [26] separated these reactions so that each product is a separate entry containing the same reactants. After removing duplicates and reactions with incorrect atom mappings, approximately 1 M unique reactions remained, which were further divided into train/valid/test sets containing approximately 800k/100k/100k reactions, respectively. This is the USPTO-full dataset. The USPTO-50k [24] dataset is a subset containing about 50*k* reactions, which are classified into 10 predefined categories. The sizes of train/valid/test are 40, 008/5, 001/5, 007 [26]. We first compare RetroRanker with a energy-based re-ranking model, rxn-ebm [23]. Note that RetroRanker encodes both molecular features and reaction change features, while only molecular features are utilized in rxn-ebm. In contrast, RetroRanker incorporates additional reaction change features at the atom- and bond-level, which we believe contribute to the improved performance. RetroRanker is a pairwise ranking model, while rxn-ebm is an energy-based ranking model. Training an energy-based model requires integrating the energy over all possible predictions to obtain meaningful probability distributions while training pairwise ranking models is more flexible. Lin et al. [23] simplified the training process of energy-based models by using up to 50 predictions. We compare RetroRanker with rxn-ebm over RetroXpert [40] and GLN [26] on the USPTO-50k dataset. RetroXpert [40] is a template-free method combining both graph-level and sequence-level features; it first predicts reaction centers and then generates synthons, which are further used to predict the reactants. GLN [26], or Graph Logic Network, is a template-based method that ranks reaction templates based on graph neural networks. For fair comparisons with rxn-ebm, we build the pairs to train RetroRanker based on the predictions released by [23].

Augmented Transformer [14] (on USPTO-full) and R-SMILES [15] (on USPTO-50k and USPTO-full) are state-of-the-art retrosynthesis approaches based on Transformers [12]. We further test RetroRanker on Augmented Transformer and R-SMILES. Augmented Transformer [14] introduces data augmentation to the original Transformer model [12]. R-SMILES [15] introduces root-aligned SMILES of the reactants and product to Augmented Transformer, significantly reducing the overall edit distance between the product and reactants SMILES, making it easier to learn the chemical knowledge for reactions. For R-SMILES, we use the released data and model 2 to obtain predictions. On USPTO-50k, the train/val/test data are augmented by 20 times, and on USPTO-full are augmented by 5 times. We obtain predictions by feeding the model with train, valid, and test data, respectively. For a given product molecule, each predicted entry is a set of reactants; we pair it with the product and obtain the mapped atoms using RXNMapper [31]. The training and valid data to train RetroRanker are constructed based on model predictions on the training and valid split from the original dataset. Both the training and valid data will be a collection of tuples, where each tuple consists of a pair of recorded reactants-product and a pair of non-recorded reactants-product. The training objective of RetroRanker is to ensure that the recorded reactants have a higher score than non-recorded predictions. During inference on the test data, we pair each prediction with the given product to obtain the RetroRanker score, which will be used during re-ranking. We train Augmented Transformer model from scratch with a large-sized model, which achieves improved results over the original paper [14]. For Augmented Transformer on USPTO-full, following [14], we add 5 augmented random SMILES to train the model; RetroRanker on Augmented Transformer is trained in a similar way as on R-SMILES. Please refer to Additional file 1: Section 2 for parameter settings of Augmented Transformer.

Table 1 shows the baselines and datasets used to evaluate RetroRanker. We choose various types of baselines mainly to demonstrate that RetroRanker is a generic re-ranking plugin that can be potentially applied to most single-step retrosynthesis models.

## Results and discussion

In this section, we first compare RetroRanker with rxn-ebm [23], an energy-based re-ranking model primarily focused on molecular features, over RetroXpert [40] and GLN [26] on the USPTO-50k dataset. We further demonstrate that our approach can achieve improved performance over the recent state-of-the-art method, R-SMILES, on the USPTO-50k dataset. Additionally, we show that RetroRanker can enhance the performance of Augmented Transformer and R-SMILES on the more challenging USPTO-full dataset. It is important to note that our re-ranking strategy is flexible, and we report the overall best results in this section. More results under different re-ranking strategies can be found in Additional file 1: Tables S3 and S4. We also integrate RetroRanker with Retro* [42] to demonstrate the effectiveness of RetroRanker in multi-step retrosynthesis planning.

### Results on USPTO-50k

**Table 2:** Top-k accuracy of rxn-ebm and RetroRanker over RetroXpert and GLN

Rank	RetroXpert			GLN		
	Original	rxn-ebm	RetroRanker*	Original	rxn-ebm	RetroRanker$^{\dag }$
Top-1	45.8 ± 0.3	42.7 ± 0.3	47.3 ± 0.7 (+4.6$^{\ddag }$)	51.7 ± 0.3	52.3 ± 0.0	52.1 ± 0.5 (-0.2)
Top-3	59.2 ± 0.3	62.0 ± 0.2	64.4 ± 0.7 (+2.4)	67.8 ± 0.4	74.9 ± 0.3	74.9 ± 0.2 (+0.0)
Top-5	63.0 ± 0.6	67.6 ± 0.1	70.3 ± 0.2 (+2.7)	75.1 ± 0.3	82.0 ± 0.2	82.7 ± 0.2 (+0.7)
Top-10	66.9 ± 0.3	73.0 ± 0.3	75.7 ± 0.2 (+2.7)	83.2 ± 0.1	88.0 ± 0.0	89.3 ± 0.2 (+1.3)
Top-20	69.9 ± 0.6	75.9 ± 0.1	77.1 ± 0.3 (+1.2)	88.9 ± 0.1	91.4 ± 0.1	92.1 ± 0.2 (+0.7)
Top-50	73.0 ± 0.7	77.3 ± 0.2	77.3 ± 0.3 (+0.0)	92.4 ± 0.1	93.0 ± 0.1	93.2 ± 0.1 (+0.2)

$^{*}$ The re-ranking strategy is $S1(90\%, 0)$ and the GNN backbone is AttentiveFP

$^{\dag }$ The re-ranking strategy is $S2(90\%, 0)$ and the GNN backbone is AttentiveFP

$^{\ddag }$ Numbers in parentheses denote the improvement over rxn-ebm. The RetroRanker models are trained based on the same single-step proposals with rxn-ebm

Table 2 shows the top-k accuracy of rxn-ebm [23] and RetroRanker over RetroXpert and GLN. We train our model using the same single-step proposals as in rxn-ebm, and the averaged results are reported. RetroRanker significantly enhances the accuracy of both RetroXpert and GLN, demonstrating the effectiveness of leveraging RetroRanker for re-ranking. On RetroXpert, RetroRanker outperforms rxn-ebm by a large margin, while on GLN, RetroRanker is comparable to or slightly better than rxn-ebm. It is worth noting that on RetroXpert, we use re-ranking strategy *S*1, while on GLN , we use strategy *S*2. The ranking strategy *S*2 shows more respect for the original ranking. Thus, *S*1 is more suitable for RetroXpert, as the original performance is relatively low.

On the proposals by RetroXpert, we performed additional experiments to verify the effectiveness of our model and features. Under backbones like WLN [43] or weave [44], the re-ranking performance is comparable with AttentiveFP. However, in the ablation study, the performance dropped significantly when removing reaction change features. These results can be found in Additional file 1: Table S5. Based on the results above, the improvement over rxn-ebm can be primarily attributed to the introduction of both molecular features and reaction change features, which are critical for learning representations of chemical reactions.

Table 3 shows the accuracy when re-ranking predictions of R-SMILES, with other methods included as references. Improving the results of R-SMILES is more challenging compared to RetroXpert or GLN. R-SMILES has relatively high accuracies in top-ranked predictions, e.g., the top 5 accuracy is $86.1\%$, while RetroRanker aims to mitigate the frequency bias by re-ranking those low-ranked predictions. This leaves us less room for improvement. As shown in Table 3, RetroRanker can still achieve improved performance over R-SMILES, e.g., when using the re-ranking strategy $S2(100\%,2)$ and Graphormer as the GNN backbone, the top 5 accuracy is improved by nearly $1\%$.

On USPTO-50k, we define the final accuracy as the accuracy at top-50 for the baseline model. R-SMILES achieves its final accuracy of $94.3\%$ at position 44, i.e., the performance cannot be further improved until the $50\text{th}$ prediction. After re-ranking with RetroRanker using re-ranking strategy $S2(100\%,2)$ and AttentiveFP as the GNN backbone, we achieve the final accuracy at position 37. This implies that if R-SMILES with RetroRanker is applied in multi-step retrosynthesis planning, the total search space at each step can be directly reduced by approximately $16\%$. It is important to note that the overall search space grows exponentially as the number of steps increases. This indicates that RetroRanker can potentially reduce the search space in multi-step retrosynthesis planning.

**Table 3:** Top-k accuracy$(\%)$ after re-ranking over R-SMILES on USPTO-50k

Models	Top-1	Top-3	Top-5	Top-10	Top-20
RetroSim	37.3	54.7	63.3	74.1	82.0
RetroXpert	50.4	61.1	62.3	63.4	63.9
GLN	52.5	69.0	75.6	83.7	89.0
LocalRetro	53.4	77.5	85.9	**92.4**	-
AT	53.5	–	81.0	85.7	-
R-SMILES	**56.0**	79.1	86.1	91.0	93.5
R-SMILES+RetroRanker$^{*}$	56.0	**79.6** (+0.5)	86.6 (+0.5)	91.8 (+0.8)	93.8 (+0.3)
R-SMILES+RetroRanker$^{\dagger }$	56.0	79.5 (+0.4)	**86.9** (+0.8)	91.5 (+0.5)	**94.0** (+0.5)

Bolded values represent the best top-k accuracies

$^{*}$ The re-ranking strategy is $S2(100\%, 2)$, the GNN backbone is AttentiveFP

$^{\dagger }$ The re-ranking strategy is $S2(100\%, 2)$, the GNN backbone is Graphormer

To further understand the improvement of RetroRanker, we perform a deep analysis based on reaction types. The reactions in USPTO-50k are classified into 10 categories [6], and their details can be found in Additional file 1: Table S1. We compare the re-ranked results mainly using accuracies at top-3 and top-5 for each reaction type because for re-ranking strategy $S2(100\%,2)$, the top-2 predictions remain unchanged. RetroRanker achieves significant improvement in two reaction types: C-C bond formation ($+1.8\%$ at top-3 and $+2.5\%$ at top-5) and Heterocycle formation ($+2.2\%$ at top-3 and $+10.0\%$ at top-5). The full results of RetroRanker on the 10 categories are shown in Additional file 1: Table S1. We consider the broken or newly constructed bonds as changed bonds, which usually reveal the changes during reactions. The average numbers of changed bonds for C-C bond formation and Heterocycle formation are 1.7 and 3.5, respectively, which are the two largest among all reaction types. Note that the average number of changed bonds on the whole USPTO-50k is 1.1. Thus, reactions of the two types have a greater degree of change than other reactions on average. C-C bond formation is usually a condensation or a coupling reaction during which multiple bonds are broken. The formation of heterocycles, in general, contains condensation reactions and other related processes such as tautomerization or aromatization, which are less common reactions in the dataset. The recorded reactants of the two types are less frequent and therefore become low-ranked predictions. The results also demonstrate that RetroRanker can effectively re-rank the predictions based on chemical features to mitigate the frequency bias in predictions of existing data-driven models.

### Results on USPTO-full

**Table 4:** Top-k accuracy $(\%)$ after re-ranking on USPTO-full

Models	Top-1	Top-3	Top-5	Top-10
RetroSim	32.8	–	–	74.1
GLN	39.3	–	–	63.7
LocalRetro	39.1	53.3	58.4	63.7
AT	47.6	62.4	66.7	70.7
AT+RetroRanker$^{*}$	48.0 (+0.4)	64.1 (+1.7)	68.5 (+1.8)	71.7 (+1.0)
AT+RetroRanker$^{\dagger }$	48.8 (+1.2)	64.7 (+2.3)	68.8 (+2.1)	71.7 (+1.0)
R-SMILES	48.9	66.5	71.8	76.8
R-SMILES+RetroRanker$^{\dagger }$	49.0 (+0.1)	67.2 (+0.7)	72.6 (+0.8)	77.3 (+0.5)

$^{*}$ The re-ranking strategy is $S2(100\%, 0)$, the GNN backbone is Graphormer, and the model is trained based on the predictions of AT

$^{\dagger }$ The re-ranking strategy is $S2(100\%, 0)$, the GNN backbone is Graphormer, and the model is trained based on the predictions of R-SMILES

Table 4 shows the accuracy when applying RetroRanker to Augmented Transformer and R-SMILES on the more challenging USPTO-full dataset. The results of both Augmented Transformer and R-SMILES are re-ranked using strategy $S2(100\%, 0)$, i.e., the final ranking is based on the sum of the original ranking and the ranking based on RetroRanker scores, which can be considered as an ensembled ranking of the original model and RetroRanker. With RetroRanker, we achieve improvements over both Augmented Transformer and R-SMILES, and the improvement of RetroRanker over Augmented Transformer is more significant than over R-SMILES because the overall accuracy of top-ranked predictions of R-SMILES is relatively high. When no top-ranked predictions are preserved, we achieve improvement on top-1 accuracy for both Augmented Transformer and R-SMILES. In particular, for Augmented Transformer, the top-1 accuracy is improved by 1.2%. Similar to USPTO-50k, RetroRanker can also help reduce the search space when applying RetroRanker to single-step models trained on USPTO-full to multi-step planning. For example, for Augmented Transformer, the accuracies of re-ranked predictions on top-6, 7, and 8 are comparable to or higher than the accuracies of the original top-8, 9, and 10. Please refer to Additional file 1: Table S4 for detailed comparisons.

For Augmented Transformer, compared to RetroRanker trained on its own predictions, the improvement is more significant when re-ranking with RetroRanker trained using the R-SMILES predictions. This improvement suggests that RetroRanker trained on R-SMILES could potentially be used as a generic ranking plugin to enhance the performance of other retrosynthesis models. Another scenario of leveraging RetroRanker is that, when developing new retrosynthesis models, RetroRanker on R-SMILES can be considered as a pretrained ranking model. To achieve further improved performance of the newly designed retrosynthesis model, we could generate its predictions on the training data (or a small amount of the training data) to finetune the pretrained ranking model. In this way, we could achieve improved performance more efficiently with the pretrain and finetune paradigm, which has been widely adopted in natural language processing. Please refer to Additional file 1: Section 6 for more results on this.

To understand the improvement of RetroRanker, we perform an analysis on product molecules for which rankings of recorded reactants are changed. For predictions of Augmented Transformer on USPTO-full, after re-ranking with $S2(100\%, 0)$, the rankings of recorded reactants for 13, 088 product molecules are improved, and more details of changing in rankings can be found in the Additional file 1: Section 7. For the entire USPTO-full dataset, the average number of changed bonds between recorded reactants and the given product is 1.4, while on the improved subset it is 1.7. This is intrinsically related to the frequency bias, because in template-free approaches, a reaction with more changed bonds means a greater degree of change on SMILES strings, and these decoding patterns are less frequent. Thus, the recorded reactants of these reactions become low-ranked predictions. RetroRanker is designed primarily based on chemical features, which can be considered as a complement to translation-based approaches. The results indicate that RetroRanker can improve the retrosynthesis performance on product molecules that are synthesized through less common reactions.

### Results on multi-step retrosynthesis planning

Designing multi-step retrosynthesis routes is always challenging as it requires an algorithm to select feasible reactants at each step. It is important to note that the search space grows exponentially as the number of steps increases. Numerous research studies attempted to reduce the search space by leveraging search algorithms such as Monte Carlo Tree Search [45] or A* algorithm [42].

RetroRanker has the potential to reduce search spaces through re-ranking. We conducted preliminary studies on leveraging RetroRanker in Retro* [42], a neural-guided A* search algorithm for multi-step retrosynthesis planning. The test set of Retro* is constructed from molecules on USPTO dataset that can be synthesized using *eMolecules*^3^ via existing reactions in the dataset. The authors trained a template-based model [46] as the single-step retrosynthesis tool in route planning and further refined the routes to ensure each reaction is covered within top-50 predictions by the single-step model. The test set contains 190 molecules as the targets of multi-step retrosynthesis route planning and is evaluated primarily using the planning efficiency metric, i.e., the success rate within 500 calls to the single-step model.

**Fig. 4:**
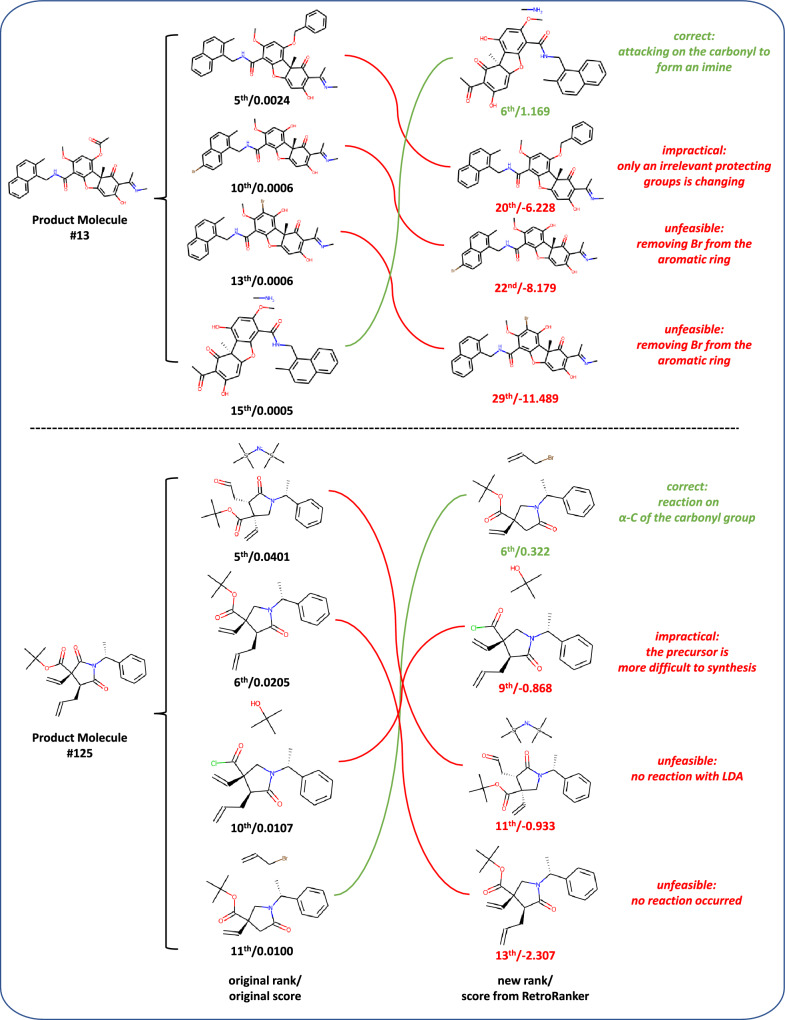
Improved predictions over Retro* leveraging RetroRanker. The recorded reactants in synthesis routes are highlighted in green. The RetroRanker scores and the re-ranked positions are shown under each prediction

We apply RetroRanker trained based on predictions of Augmented Transformer on USPTO-full to Retro*. As it is difficult to fully integrate RetroRanker with Retro* during each search step, we only use RetroRanker to re-rank predictions for the first step. After re-ranking,^4^ the success rate on the Retro* test set improves by $2.1\%$, and the average number of calls to the single-step model decreases from 156.58 [42] to 153.26. It is worth noting that likelihoods of low-ranked predictions from the single-step model are comparable, while it is critical to select chemically feasible reactants for multi-step route planning. We present two cases in Fig. 4 to demonstrate the improved performance achieved with RetroRanker. We leave the deep integration of RetroRanker in multi-step retrosynthesis planning as our future work, which requires the joint optimization of the search algorithm and the re-ranking model [47].

In multi-step retrosynthesis planning, the predicted reactants should be easier to synthesize from the building blocks than the given product. The synthesis route should not contain chemically unfeasible reactants, e.g., reactions that will not occur at all. The two cases shown in Fig. 4 are failed cases in Retro* using the original single-step model, while correct synthesis routes can be obtained after re-ranking with RetroRanker only in the first step.

In the first product molecule (#13 in the original dataset), multiple functional groups are presented, and the single-step model generates up to 29 predictions for the initial step. The scores for low-ranked reactants are quite low. Many of these low-ranked reactants are chemically unfeasible; for example, the $10\text{th}$ and the $13\text{th}$ reactants attempt to remove the Br atom from the aromatic ring. These reactants are re-ranked to lower positions with relatively low scores from RetroRanker. The $5\text{th}$ potential reaction tries to change the protecting group on the phenol hydroxyl, which does not aid in further dividing the molecule into synthesizable building blocks. Occasionally, it is necessary to add protecting groups before condensation or coupling reactions, but it is not the case for this molecule; thus, the $5\text{th}$ prediction is also re-ranked to a lower position. The RetroRanker score of the $15\text{th}$ prediction is relatively high, representing an addition-elimination reaction where a methylamine attacks the carbonyl group to form an imine. This reaction is chemically feasible and simplifies the target molecule, resulting in its re-ranking to a higher position.

For the second case (#125), RetroRanker eliminates chemically unreactive predictions. The $5\text{th}$ predicted reactants include a molecule similar to the product molecule and a strong base diisopropylamide (usually generated from the lithium diisopropylamide/LDA). However, no reaction occurs under the given substrate and condition, indicating that the reaction template for this prediction may be incorrectly applied. Similarly, the $6\text{th}$ reactants contain a molecule that has one fewer carbonyl group than the product molecule, which is unfeasible. These incorrect reactants are common in data-driven retrosynthesis approaches. The templates only capture the local environment for reactions, which is insufficient. RetroRanker encodes molecular features and reaction change features with GNNs, allowing for better representation of the reaction and the capability to filter out chemically unfeasible predictions. In this case, the two incorrect reactants receive relatively low scores and are re-ranked to the $11\text{th}$ and $13\text{th}$ positions, respectively. We also present a possible but impractical reactant. The $10\text{th}$ prediction is an esterification reaction on the acyl chloride; however, synthesizing the acyl chloride is more challenging than the product molecule, rendering this step unsuitable for a retrosynthesis route. The substitution reaction at the $11\text{th}$ position is re-ranked to a higher position, as it attempts to synthesize the allyl group outside the ring, significantly simplifying the structure.

Through these cases, we can observe that RetroRanker effectively re-ranks predictions, assigning chemically reasonable scores. After re-ranking, the search algorithm is more likely to identify the correct reactants, thereby boosting the performance in multi-step retrosynthesis planning.

## Conclusion

We propose RetroRanker, a re-ranking model built upon graph neural networks, to mitigate frequency bias in the predictions of state-of-the-art data-driven approaches in single-step retrosynthesis prediction. We incorporate both molecular features and reaction change features as chemical features into GNNs, and achieve improved performance over existing approaches by re-ranking low-ranked predictions. Our preliminary study also demonstrates that RetroRanker can reduce the search space in multi-step retrosynthesis. As RetroRanker can be flexibly applied to most existing single-step retrosynthesis models, we believe it holds great potential for widespread use in future retrosynthesis analysis studies.

## Supplementary Information

**Additional file 1.** The model details, training settings and supplementary results are provided in the Additional material.

## Data Availability

The dataset and source code used in this paper are publically available at https://github.com/catalystforyou/RetroRanker. The predictions used in rxn-ebm can be found at https://github.com/coleygroup/rxn-ebm/.

## Footnotes

1 We use the open-source tool AiZynthFinder, https://github.com/MolecularAI/aizynthfinder.

2 https://github.com/otori-bird/retrosynthesis.

3 http://downloads.emolecules.com/free/2019-11-01/.

4 The re-ranking strategy is $S1(80\%, 4)$.
