# Supplementary material for: RetroRanker: leveraging reaction changes to improve retrosynthesis prediction through re-ranking
Source: J Cheminform. 2023 Jun 8;15:58. doi: 10.1186/s13321-023-00727-7 (PMC10249296; doi:10.1186/s13321-023-00727-7)
Supplement: Supplementary file 1 — Additional file 1. The model details, training settings and supplementary results are provided in the Additional material. [file 13321_2023_727_MOESM1_ESM.pdf]

Supplementary Material for RetroRanker: Leveraging Reaction Changes to Improve Retrosynthesis Prediction through Re-ranking

Junren Li, Lei Fang, Jian-Guang Lou

1 Features used in RetroRanker

We show the complete list of features and their sizes below.

Node features (106)

Atom features (57):

- Atom type (15, one-hot): H, B, C, Si, N, O, F, As, Se, P, S, Cl, Br, I, unk;
- Chiral type (2, one-hot): clockwise, counterclockwise;
- Hybridization (5, one-hot): SP, SP2, SP3, SP3D, SP3D2
- Degree (6, one-hot): 0, 1, 2, 3, 4, 5;
- Explicit valence (5, one-hot): 0, 1, 2, 3, 4;
- Implicit valence (5, one-hot): 0, 1, 2, 3, 4;
- Formal charge (6, one-hot): -2, -1, 0, 1, 2, 3;
- Aromatic (1, Bool): True/False;
- Total number of H (5, one-hot): 0, 1, 2, 3, 4;
- Is H-bond donor (1, bool): True/False;
- Is H-bond acceptor (1, bool): True/False;
- Is in an n-number ring (6, one-hot), the values of n: 3, 4, 5, 6, 7, 8;

Change features (49):

- Number of small-sized reactants [1] (4, one-hot): number of reactants whose atoms are less than half of the product, 0, 1, 2, 3;
- Number of medium-sized reactants (4, one-hot): number of reactants whose atoms are more than half, but less than the whole of the product, 0, 1, 2, 3;
- Number of large-sized reactants (2, one-hot): number of reactants that have more atoms than the product, 0, 1;
- Is reacted atom (1, bool): True/False;
- Is atom of leaving groups (1, bool): True/False;
- Total number of reacted atoms (5, one-hot): 0, 1, 2, 3, ≥4;
- Total number of atoms in leaving groups (7, one-hot): 0, 1, 2, 3, 4, 5, ≥6;

[1] The sizes of the reactant molecules provide hints to infer reaction types. For instance, small-sized reactants usually appear in addition or substitution reactions, while a reaction with medium-sized reactants is more likely to be a coupling reaction, and a large-sized reactant can be a substrate in deprotection reactions.

- Number of reacted atoms in neighborhoods (5, one-hot): 0, 1, 2, 3, ≥4;
- Number of leaving atoms in neighborhoods (5, one-hot): 0, 1, 2, 3, ≥4;
- Number of changed C-C bonds in neighborhoods (3, one-hot): 0, 1, 2;
- Number of changed C-X bonds in neighborhoods (3, one-hot): 0, 1, 2;
- Number of changed X-X bonds in neighborhoods (3, one-hot): 0, 1, 2;
- Number of changed double bonds in neighborhoods (3, one-hot): 0, 1, 2;
- Number of changed aromatic bonds in neighborhoods (3, one-hot): 0, 1, 2;

Edge features (13)
Bond features (12)

- Bond type (4, one-hot): single, double, triple, aromatic;
- Conjugated (1, bool): True/False;
- Aromatic (1, bool): True/False;
- Stereo (6, one-hot): none, any, Z, E, cis, trans.

Self-loop (1)

- Is self-loop edge (1, bool): True/False

2 Training setting

2.1 Training Settings of Augmented Transformer

We re-trained Augmented Transformer on the USPTO-full dataset. For each instance, we introduce 5 random SMILES as data augmentation to the Transformer model. We train Augmented Transformer model mainly following the settings of MolecularTransformer [2], the difference is that we use 8 transformer layers and the hidden size is changed to 512. The model is trained on 8x Nvidia V100 32G GPU for about 120,000 steps. Our re-trained model outperforms the original Augmented Transformer [1], mainly because the model size is much larger.

2.2 RetroRanker with the AttentiveFP

We use two independent three-layer AttentiveFP networks to encode the reactant and product molecular graphs. The size of hidden features is 512 and the dropout is 0.2. The reaction representation is obtained by concatenating six readout channels on molecular graphs or masked molecular graphs, namely, the reactant molecular graph, the product molecular graph, the reactant molecular graph with reacted atoms as masks, the reactant molecular graph with atoms in the leaving groups as masks, the product molecular graph with reacted atoms as masks, and the bond information in reactant molecular graphs. The masked graphs also reveal potential reaction changes, where node representations correspond to masked atoms are preserved, while other node representations are set to $\mathbf{0}$. We use a two linear-layer neural network with reaction representations as input to obtain RetroRanker score for each reactants-product pair. RetroRanker is trained using the label smoothed (0.01) cross-entropy loss function. We use Adam [2] optimizer with a initial learning rate of 3e-4 and a weight decay of 1e-5. The batch size is 512. To prevent overfitting, we use valid data to early stop the training process. On USPTO-50k, we trained the model with an Nvidia A100 80G GPU and it takes approximately 12 hours, while on USPTO-full is 40 hours.

2.3 RetroRanker with Graphormer

Similar to RetroRanker with AttentiveFP, we use two independent Graphormer encoders to encode the reactant and product molecular graphs. The settings of the Graphormer encoder follow the "graphormer_base" [3] architecture. The reaction representation is obtained by concatenating the rep-

[2] https://github.com/pschwllr/MolecularTransformer#training
[3] https://github.com/microsoft/Graphormer/blob/main/graphormer/models/graphormer.py

Table 1: The number of reactions and the re-ranking results for each type on USPTO-50k.

Reaction Type	On whole dataset	On test set	Top-3 Acc.†	Top-5 Acc.†
Heteroatom alkylation and arylation	15122	1516	78.4%(78.6%)	87.4%(86.9%)
Acylation and related processes	11913	1190	89.1%(89.6%)	92.9%(93.1%)
C-C bond formation	5639	567	59.8%(61.6%)	70.2%(72.7%)
Heterocycle formation	900	90	67.8%(70.0%)	71.1%(81.1%)
Protections	650	68	86.8%(83.8%)	91.2%(89.7%)
Deprotections	8353	824	76.1%(76.7%)	81.2%(83.4%)
Reductions	4585	462	80.1%(79.7%)	86.4%(87.8%)
Oxidations	814	82	76.8%(79.3%)	85.4%(86.6%)
Functional Group Interconversion (FGI)	1834	183	55.2%(53.6%)	65.6%(64.5%)
Functional Group Addition (FGA)	227	23	87.0%(91.3%)	91.3%(91.3%)

† The accuracy is based on R-SMILES' prediction. The number in parentheses is reranked with $S2(100\%, 2)$.

resentation of "CLS" token from the reactant and the product, where "CLS" is a special token in Transformers-based models. On USPTO-full, we train the model with 500,000 steps on 8x Nvidia V100 32G GPUs. We report the results using the averaged model parameters of the last 10 checkpoints. On USPTO-50k, the model is trained with 50,000 steps. We report the results using the averaged model parameters of the last 5 checkpoints.

3 Reactions on USPTO-50k

Supplementary Table 1 shows the number of reactions and the re-ranking results for each type on the USPTO-50k dataset.

4 Results of re-ranking strategies under different parameters and predictions.

As shown in Table 4, RetroRanker achieves the best performance when re-ranking predictions with strategy $S2$, i.e., $S2(100\%, 2)$ on USPTO-50k. We find that the overall result of using re-ranking strategy $S2$ is comparable under different re-ranking ratios, provided that the re-ranking ratio p is above a certain threshold (e.g., 75%).

We mainly show the effect of the re-ranking parameters for the strategy $S1$ using RetroRanker over Augmented Transformer. Supplementary Figure 1 shows the accuracies when varying the re-ranking ratio (Suplementary Figure 1(a)) and the number of preserved top-ranked predictions (Supplementary Figure 1(b)). In Figure 1(a), when increasing the re-ranking ratio p, the performance is also improved. The overall results are comparable when the re-ranking ratio p is above 0.75. This also explains that the results of using $S2$ are comparable when the re-ranking ratio is above the threshold of 0.75. Figure 1(b) shows that RetroRanker achieves improved performance when varying the number of preserved top-ranked predictions. One interesting observation is that, if the top-1 prediction is preserved, the accuracy of the top-2 is the best among all settings, similarly, if the top-3 predictions are preserved, the accuracy of the top-4 is the best. This indicates that our re-ranking strategy is flexible, we can tune the number of preserved top-ranked predictions for certain performance requirements, e.g., if we would like to have the best overall accuracy at top-20, we can set the number of preserved predictions to 18 or 19.

We show an illustrative example in Supplementary Table 2 to describe the re-ranking process. Note that only predictions whose RetroRanker scores are among the bottom 50% are re-ranked in $S1(50\%, 3)$, while the ranking of other predictions are preserved.

5 Results on various GNN backbones and strategies

Supplementary Table 3 and 4 show the full results of re-ranking on USPTO-50k and USPTO-full, respectively. With re-ranking strategy $S2(100\%, 2)$, on USPTO-50k, RetroRanker with Graphormer

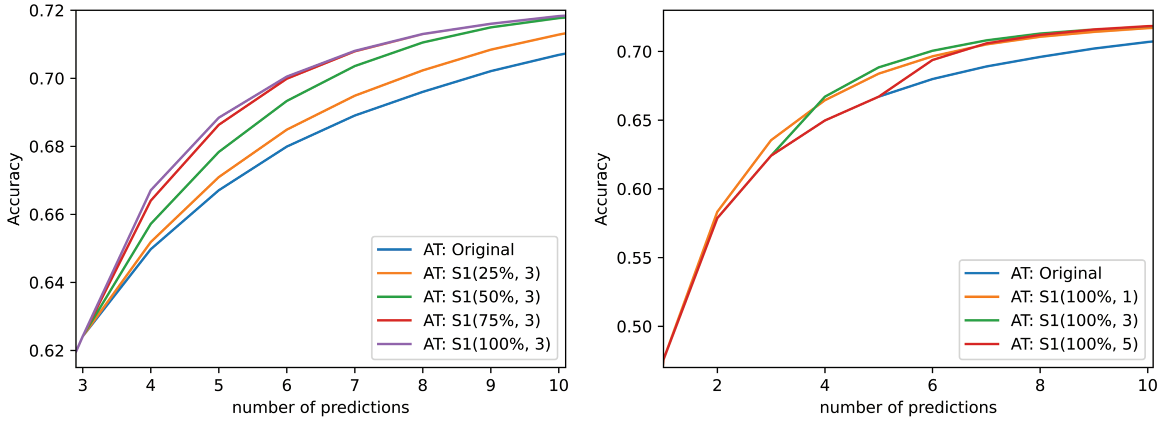

Figure 1: The improvement under different parameters of re-ranking strategy $S1$ for Augmented Transformer. (a) The accuracies under different re-ranking ratios. (b) The accuracies under different numbers of preserved predictions.

Table 2: An illustrative example of changed rankings after re-ranking

Original rank	RetroRanker score	$S1(50\%, 3)^*$	$S1(100\%, 3)^\dagger$	$S2(100\%, 3)$
1	+0.5	1	1	1 (1+1)‡
2	-2.0	2	2	2 (2+2)
3	-3.0	3	3	3 (3+3)
4	-1.5	8	8	5 (4+8)
5	-1.0	4	7	6 (5+7)
6	-0.5	5	6	7 (6+6)
7	+1.0	6	4	4 (7+4)
8	-3.5	9	9	9 (8+9)
9	+0.0	7	5	8 (9+5)
10	-4.0	10	10	10 (10+10)

* The top-3 predictions are preserved in $S1(50\%, 3)$. Predictions whose RetroRanker scores are among the bottom 50% are highlighted in red. They are moved to the end of the list and will be re-ranked based on RetroRanker scores. The order of other predictions (highlighted in green) is preserved.

† The top-3 predictions are preserved in $S1(100\%, 3)$. All other predictions are re-rank based on RetroRanker scores (highlighted in red).

‡ Numbers in parentheses denote the sum of the original rank and new rank in $S1(100\%, 3)$.

improves all the top-5 to top-9 accuracies by nearly 1%, and RetroRanker with AttentiveFP also improves the top-7 to top-10 accuracies by about 1%. On USPTO-full, the best improvement on Augmented Transformer is achieved using strategy $S2(100\%, 0)$ with Graphormer, which improves the top-3 to top-5 accuracy by more than 2%. Note that the results we report here are all trained based on predictions of R-SMILES. We tune the parameters (re-ranking ratio and the number of preserved predictions) on R-SMILES under $S1$ strategy, and find that it is difficult to achieve steady improvement. When using the strategy $S2(100\%, 0)$, we achieve improvement over all positions, and RetroRanker with Graphormer performs better than that with AttentiveFP. For example, the top-4 to top-7 accuracies are improved by nearly 1%.

Table 3: Top-k accuracy(%) after re-ranking on USPTO-50k with various GNN backbones and strategies

	Top-3	Top-4	Top-5	Top-6	Top-7	Top-8	Top-9	Top-10
R-SMILES	79.1	83.6	86.1	87.8	88.8	89.6	90.2	91.0
+RetroRanker(AF)*	79.4(+0.3)	83.9(+0.3)	86.3(+0.2)	88.2(+0.4)	89.1(+0.3)	90.0(+0.4)	90.7(+0.5)	91.4(+0.4)
+RetroRanker(AF)†	79.7(+0.6)	83.9(+0.3)	86.5(+0.4)	88.2(+0.4)	89.7(+0.9)	90.7(+1.1)	91.4(+1.2)	91.8(+0.8)
+RetroRanker(GH)*	79.4(+0.3)	83.8(+0.2)	86.4(+0.3)	88.3(+0.5)	89.3(+0.5)	90.0(+0.4)	90.7(+0.5)	91.1(+0.1)
+RetroRanker(GH)†	79.5(+0.4)	84.0(+0.4)	86.9(+0.8)	88.5(+0.7)	89.5(+0.7)	90.5(+0.9)	91.0(+0.8)	91.5(+0.5)

* The re-ranking strategy is $S1(50\%, 2)$. "AF" is for AttentiveFP, and "GH" is for Graphormer, the abbreviations are also used in following tables.
† The re-ranking strategy is $S2(100\%, 2)$.

Table 4: Top-k accuracy(%) after reranking on USPTO-full with various GNN backbones and strategies

	Top-3	Top-4	Top-5	Top-6	Top-7	Top-8	Top-9	Top-10
AT	62.4	65.0	66.7	68.0	68.9	69.6	70.2	70.7
+RetroRanker(AF)*	64.0(+1.6)	67.1(+2.1)	68.8(+2.1)	70.0(+2.0)	70.7(+1.8)	71.2(+1.6)	71.5(+1.3)	71.8(+1.1)
+RetroRanker(AF)†	64.4(+2.0)	67.1(+2.1)	68.8(+2.1)	69.9(+1.9)	70.7(+1.8)	71.2(+1.6)	71.6(+1.4)	71.8(+1.1)
+RetroRanker(GH)*	64.2(+1.8)	67.1(+2.1)	68.8(+2.1)	69.8(+1.8)	70.4(+1.5)	70.9(+1.3)	71.3(+1.1)	71.5(+0.8)
+RetroRanker(GH)†	64.7(+2.3)	67.2(+2.2)	68.8(+2.1)	69.8(+1.8)	70.6(+1.7)	71.1(+1.5)	71.4(+1.2)	71.7(+1.0)
R-SMILES	66.5	69.7	71.9	73.4	74.5	75.4	76.1	76.7
+RetroRanker(AF)‡	66.6(+0.1)	69.8(+0.1)	71.8(-0.1)	73.0(-0.4)	74.0(-0.5)	74.6(-0.8)	75.2(-0.9)	75.7(-1.0)
+RetroRanker(AF)△	66.6(+0.1)	69.8(+0.1)	71.9(+0.0)	73.5(+0.1)	74.6(+0.1)	75.5(+0.1)	76.3(+0.2)	76.9(+0.2)
+RetroRanker(GH)‡	66.7(+0.2)	70.1(+0.5)	72.3(+0.5)	73.7(+0.4)	74.7(+0.2)	75.5(+0.1)	76.1(-0.1)	76.6(-0.1)
+RetroRanker(GH)†	67.2(+0.7)	70.4(+0.8)	72.6(+0.8)	74.1(+0.8)	75.2(+0.7)	76.1(+0.7)	76.8(+0.6)	77.3(+0.6)

* The re-ranking strategy is $S1(100\%, 2)$.
† The re-ranking strategy is $S2(100\%, 0)$.
‡ The re-ranking strategy is $S1(50\%, 2)$.
△ The re-ranking strategy is $S2(100\%, 2)$.

On proposals predicted by RetroXpert, we performed additional experiments to verify the effectiveness of our model and features. Under backbones like WLN [3] or weave [4], the re-ranking performance is comparable with AttentiveFP. However, in the ablation study, the performance dropped significantly when removing change features, as shown in Supplementary Table 5. The improvement over `rxn-ebm` can be primarily attributed to the introduction of both molecular features and reaction change features, which are crucial for learning the representations of chemical reactions.

Table 5: Ablation studies over various GNN backbones and reaction change features

Model	Top-1	Top-3	Top-5	Top-10	Top-20	Top-50
RetroXpert	45.8 ± 0.3	59.2 ± 0.3	63.0 ± 0.6	66.9 ± 0.3	69.9 ± 0.6	73.0 ± 0.7
+ `rxn-ebm`	42.7 ± 0.3	62.0 ± 0.2	67.6 ± 0.1	73.0 ± 0.3	75.9 ± 0.1	77.3 ± 0.2
+ RetroRanker(AF)*	47.3 ± 0.7	64.4 ± 0.7	70.3 ± 0.2	75.7 ± 0.2	77.1 ± 0.3	77.3 ± 0.3
+ RetroRanker(WLN)*	47.3 ± 0.6	64.4 ± 0.6	70.3 ± 0.4	75.8 ± 0.1	77.1 ± 0.3	77.3 ± 0.3
+ RetroRanker(Weave)*	47.3 ± 0.6	64.4 ± 0.4	70.3 ± 0.2	75.5 ± 0.1	77.2 ± 0.3	77.3 ± 0.3
+ RetroRanker(AF, w/o change features)*	46.3 ± 0.6	61.7 ± 0.7	66.3 ± 0.5	70.9 ± 0.6	75.8 ± 0.3	77.3 ± 0.3

* The re-ranking strategy is $S1(90\%, 0)$.

6 Re-ranking Augmented Transformer predictions with Retro-Ranker trained over R-SMILES

We find that for Augmented Transformer, compared to RetroRanker trained on its own predictions, the improvement is more significant when re-ranking with RetroRanker trained using the R-SMILES data. Suppplementary Table 6 shows the results on re-ranking with RetroRanker trained under various settings. The results show that RetroRanker trained on R-SMILES can potentially be considered as a plug-and-play re-ranking plugin, or a pretrained ranking model that can be finetuned, to achieve improved performance.

Table 6: Top-k accuracy(%) on USPTO-full with RetroRanker under various settings

Models	Top-1	Top-3	Top-5	Top-10
AT	47.6	62.4	66.7	70.7
+RetroRanker(AF, AT)[†]	48.0 (+0.4)	64.4 (+2.0)	68.8 (+2.1)	71.8 (+1.1)
+RetroRanker(AF, R-SMILES)	47.3 (-0.3)	63.6 (+1.2)	68.0 (+1.3)	71.4 (+0.7)
+RetroRanker(GH, AT)	48.0 (+0.4)	64.1 (+1.7)	68.5 (+1.8)	71.7 (+1.0)
+RetroRanker(GH, R-SMILES)	48.8 (+1.2)	64.7 (+2.3)	68.8 (+2.1)	71.7 (+1.0)

[†] RetroRanker(X, Y) denotes that the re-ranking model is trained on the predictions of model Y with X as the GNN backbone, e.g., RetroRanker(AF, AT) means that the RetroRanker model is trained based on predictions of Augmented Transformer using the AttentiveFP as GNN backbone. The re-ranking strategy used in this table is $S2(100\%, 0)$.

7 Analysis on ranking after RetroRanker for Augmented Transformer's predictions

For predictions of Augmented Transformer on USPTO-full, after re-ranking with $S2(100\%, 0)$, the rankings of recorded reactants for $13,088$ product molecules are improved, while there are also $5,527$ recorded reactants experienced a decline. The average increase in ranking is 2.1, while the average decline in ranking is 1.7. This indicates that the overall improvement in ranking is more significant than the observed decline, as the extent of their improvement is greater than that of the reactants with decreased rankings.

References

[1] Igor V Tetko, Pavel Karpov, Ruud Van Deursen, and Guillaume Godin. State-of-the-art augmented nlp transformer models for direct and single-step retrosynthesis. *Nature communications*, 11(1):1–11, 2020.

[2] Diederik P Kingma and Jimmy Ba. Adam: A method for stochastic optimization. *arXiv preprint arXiv:1412.6980*, 2014.

[3] Wengong Jin, Connor Coley, Regina Barzilay, and Tommi Jaakkola. Predicting organic reaction outcomes with weisfeiler-lehman network. *Advances in neural information processing systems*, 30, 2017.

[4] Steven Kearnes, Kevin McCloskey, Marc Berndl, Vijay Pande, and Patrick Riley. Molecular graph convolutions: moving beyond fingerprints. *Journal of computer-aided molecular design*, 30:595–608, 2016.
